# Cyclin dependent kinase 9 inhibitor induces transcription-replication conflicts and DNA damage accumulation in breast cancer

**DOI:** 10.1186/s12935-025-03897-6

**Published:** 2025-07-25

**Authors:** Minyoung Lee, Kyung-Hun Lee, Ahrum Min, So Hyeon Kim, Sujin Ham, Hae Min Hwang, Youlim Noh, Yu-Jin Kim, Dae-Won Lee, Jiwon Koh, Seock-Ah Im

**Affiliations:** 1 Cancer Research Institute, Seoul National University, Seoul, Republic of Korea; 2 Biomedical Research Institute, Seoul National University Hospital, Seoul, Republic of Korea; 3 Interdisciplinary Programs in Cancer Biology Major, Seoul National University Graduate School, Seoul, Republic of Korea; 4 Department of Internal Medicine, Seoul National University Hospital, Seoul National University College of Medicine, Seoul, Republic of Korea; 5 Department of Translational Medicine, Seoul National University College of Medicine, Seoul, Republic of Korea; 6 Department of Pathology, Seoul National University Hospital, Seoul National University College of Medicine, Seoul, Republic of Korea

**Keywords:** Breast cancer, Cyclin dependent kinase 9, DNA damage, Transcription-replication conflict

## Abstract

**Background:**

Cyclin-dependent kinase 9 (CDK9) is a crucial regulator of transcriptional progression of RNA polymerase-II (RNAP2). RNA polymerases trapped in DNA can be a source of transcription-replication conflict (T-R conflict), which is a common source of replication stress. AZD4573, a highly selective CDK9 inhibitor, has been shown to induce apoptosis in leukemia cell lines, while its anti-tumor potential in breast cancer has yet to be elucidated.

**Methods:**

To evaluate the cytotoxicity of AZD4573 in vitro, MTT assays were performed. The expression of signal transduction molecules was determined using Western blotting, immunoprecipitation, and immunofluorescence. Apoptotic cell death was verified by the annexin-V assay. DNA strand breaks and repair efficacy were evaluated through the alkaline comet assay. The siRNA knock-down system was used to confirm the action mechanism.

**Results:**

AZD4573 induced T-R conflicts during S-phase, increasing replication stress and DNA strand breaks, resulting in apoptosis by induction of caspase-3. Furthermore, we identified Dead-box 25 (DDX25) helicase as a key mediator in resolving the T-R conflicts. Nuclear translocation of DDX25 correlated with reduced sensitivity to AZD4573 by the resolution of T-R conflicts.

**Conclusions:**

Inhibition of CDK9 by AZD4573 induces the accumulation of DNA damage through T-R conflicts. DDX25 helicases were identified as a key mediator in resolving T-R conflicts and the reduced sensitivity to AZD4573.

**Supplementary Information:**

The online version contains supplementary material available at 10.1186/s12935-025-03897-6.

## Background

Breast cancer is the most common type of cancer in women, contributing significantly to mortality and morbidity [1]. Despite substantial progress in the therapeutic agents over the past few decades, which include chemotherapy, endocrine therapy, targeted therapy, and immunotherapy, the heterogeneous nature of breast cancer and its resistance to current treatments underscore the persistent need for novel therapeutic approaches [2].

Cyclin-dependent kinases (CDKs) are a family of serine/threonine protein kinases that are activated by regulatory subunits, known as cyclins, forming heterodimeric complexes. CDKs have important roles in the control of the cell cycle and modulating gene transcription. Distinct CDKs (CDK7, CDK8, CDK9, CDK12, CDK13, CDK19) are involved in different transcriptional phases of initiation, elongation, and termination [3–7]. CDK9 is a crucial regulator of transcriptional progression. Through phosphorylation of serine-2 within the c-terminal domain (CTD) of RNA polymerase II (RNAP2) and negative elongation factor (NELF), CDK9 enables the release of the paused RNAP2 from the initiation site and permits subsequent elongation [8–10]. Therefore, inhibition of CDK9 results in trapped RNAP2, leading to transient transcriptional suppression, downregulation of gene expression, and initiation of transcription-replication conflict (T-R conflict) [11–14].

T-R conflicts are considered profound threats to genome stability. Normally, transcription coordinates with replication to minimize the possibility of conflicts. Despite this, T-R conflicts can occur in aberrant transcription and dysregulated cell cycle conditions [15–19]. Mechanistically, T-R conflicts transiently form DNA-RNA hybrid structures, where nascent RNA hybridizes with the complementary DNA strand. DNA-RNA hybrids induce R-loop accumulation and replication fork stalling by disruption of DNA polymerase progression, and consequently replication stress and genomic instability [13, 17, 20, 21]. To preserve genome integrity, cells have developed multiple pathways to eliminate R-loops. RNase H enzymes precisely cleave the RNA strand in DNA-RNA hybrids and prevent excessive R-loops [22, 23]. While topoisomerase 1 (TOP1) resolves DNA supercoiling and suppresses the accumulation of DNA-RNA hybrids [24, 25]. RNA splicing factors, serine/arginine-rich splicing factor1 (SRSF1), protect the nascent RNA from annealing to the template DNA strand [26], and the transcription-export (TREX) complex, which includes the THO complex, promotes the nuclear export of messenger RNA [27, 28]. In addition, many helicases have been reported to unwind DNA and RNA molecules and play a critical role in R-loop biogenesis. SETX (senataxin), BLM (Bloom syndrome), WRN (Werner syndrome), and FANCM (Fanconi anemia complementation group M) are identified to possess proficient abilities for unwinding DNA-RNA hybrids. Recent studies have demonstrated that DEAD-box (DDX) helicases such as DDX5, DDX17, and DDX41 are essential for R-loop resolution and have been extensively studied as key markers in this process [29–36].

AZD4573 is a potent CDK9 inhibitor with nanomolar potency and selectivity over other CDK members. Previous studies have reported that AZD4573 induces cell death through down-regulation of anti-apoptotic molecules, such as MCL1, and shows a significant anti-tumor effect in acute myeloid leukemia in vitro and in vivo [37, 38]. The anti-tumor effect of AZD4573 has not been widely investigated in breast cancer or other solid cancers. The crucial roles of CDK9 in T-R conflicts and DNA damage, leading to genomic instability, prompted us to investigate the efficacy of AZD4573 in breast cancer. Furthermore, we aimed to elucidate the action mechanism of AZD4573 and identify potential biomarkers to predict the responses.

## Methods

### Reagents

AZD4573 was kindly provided by AstraZeneca (Macclesfield, Cheshire, UK) and reconstituted in dimethyl sulfoxide (DMSO) to prepare a 10 mmol/L stock solution. Z-VAD-FMK was purchased by MedChemExpress (Monmouth Junction, NJ, USA) and was initially dissolved in dimethyl sulfoxide (DMSO). Compounds were stored at – 80 ℃.

### Cell line and culture

Human breast cancer cells, MDA-MB-453, MDA-MB-231, HCC1954, HCC1143, HCC38, HCC70, HCC1419, HCC2218, HCC1395, HCC1937, HCC1428, BT-474, SK-BR-3, MCF7, T47D, KPL-4, and ZR-75–1 were obtained from the Korean Cell Line Bank (KCLB, Seoul, Republic of Korea). MDA-MB-157, MDA-MB-468, BT-549, and Hs578T were purchased from American Type Culture Collection (ATCC, Manassas, VA, USA) and authenticated by short tandem repeat analysis. Cells were cultured at 37 ℃ and 5% CO_2_ in RPMI-1640 (Welgene, Inc., Daegu, Republic of Korea) supplemented with 10% fetal bovine serum (FBS) (Gibco, Thermo Fisher Scientific Inc., Waltham, MA, USA) and 10 μg/mL gentamicin (Cellgro, Manassas, VA, USA).

### Cell viability assay

Cells were seeded in 96-well plates at a density of 2–10 × 10^3^ cells per well in 100 μL of culture medium and were incubated for 24 h at 37 ℃ to allow adherence. The cells were then treated with AZD4573 at serial concentrations (5, 10, 15, 20, 25, 50, 100 nmol/L) for 48 h. After drug treatment, 3-(4,5-dimethylthiazol −2-yl)−2,5-diphenyltetrazolium bromide solution (MTT) was added to each well, and absorbance was measured at 540 nm using a Multiskan GO Microplate spectrophotometer (Thermo Fisher Scientific Inc.). The IC_50_ was calculated using Sigma Plot software (Statistical Package for the Social Sciences, Inc., Chicago, IL, USA) [39].

### Real-time PCR

RNA was extracted using Trizol reagent (Molecular Research Center, Montgomery, OH, USA, #TR 118) according to the manufacturer’s instructions. 6 μg of total RNA was used for cDNA synthesis with random hexamers. mRNA expression levels were analyzed by StepOne™ Real-Time PCR (Thermo Fisher Scientific Inc.). The mRNA expression value was normalized to actin expression levels after measuring the signal intensities using ImageJ software (version 1.54p; National Institutes of Health, Bethesda, MD, USA). The following primer sequences were used:

CDK9, 5′-AGTACGACTCGGTGGAGTC-3′, 3′-TGTAATGGGGAACCCCTCCT-5′; MYC, 5′-TCTACACTAACATCCCACGCT-3, 3′-AATCATCGCAGGCGGAACAG-5′; Actin, 5′-AGAGCTACGAGCTGCCTGAC-3′, 3′-GGATGCCACAGGACTCCA-5′

### Western blot analysis

For whole-cell extraction, cells were washed in ice-cold PBS and collected with 1700 rpm centrifugation for 7 min at 4 ℃. To prepare the cytoplasm/nucleus subcellular fraction, cells were collected and fractionated using a subcellular protein fractionation kit (Thermo Fisher Inc., #78840) following the manufacturer’s instructions.

The following antibodies were used: phospho-RNAP2 (Ser2) was purchased from BioLegend (San Diego, CA, USA, #920204). Actin (AC-40) was supplied from Sigma Aldrich (St. Louis, MO, USA, #A3853). CHK1 (FL-476), Dead-box (DDX) 5, DDX25, and DDX41 were purchased from Santa Cruz Biotechnology (Santa Cruz, CA, USA, #sc-7898, #sc-166167, #sc-271730, #sc-166225). PARP was purchased from BD Biosciences (San Jose, CA, USA., #556494). CDK9 (C12F7), phospho-H2AX (ser139), MCL1 (D5V5L), Caspase-3, and phospho-CHK1 (ser345, 133D3) were purchased from Cell Signaling Technology (Danvers, MA, USA, #2316, #9718, #39224, #9661, #2348). DDX19 was purchased from BETHYL laboratories (Montgomery, TX, USA, #A300-546A).

### Immunofluorescence assay

Cells were grown on poly-L-lysine (Gibco, #P4707) coated coverslips. After 24 h, the cells were treated with AZD4573 for 3 h. After blocking, cells were stained with DNA-RNA hybrid antibodies (Clone S9.6, Millipore; Billerica, MA, USA, #MABE1095) for 2 h at 37℃. Coverslips were incubated with Alexa Fluor secondary antibodies and counterstained with DAPI (Thermo Fisher Inc., #D3571) for 3 min at room temperature and then mounted onto the slide using VECTASHIELD Mounting Media (Vector Laboratories, Burlingame, CA, USA, #H-1000–10). S9.6 positive nuclei were visualized and counted using a Zeiss LSM 800 laser scanning microscope (Carl Zeiss, Jena, Germany).

### EdU incorporation assay

Cells were plated on poly-L-lysine-coated coverslips. Cells were treated with AZD4573 for 3 h with 10 μM EdU. Next, cells were fixed in 3.7% formaldehyde. After fixation, cells were permeabilized with 0.5% Triton X-100 in PBS. Prepared Click-it reaction cocktail using the manufacturer’s protocols was added to cells (Thermo Fisher Inc., #C10419). Cells were stained with phospho-H2AX (ser139) antibodies (Cell Signaling Technology) for 2 h at 37 ℃ and incubated with Alexa Fluor secondary antibodies (Cell Signaling Technology, #A1101) and counterstained with DAPI for 3 min at room temperature. Coverslips were then mounted onto the slide using VECTASHIELD Mounting Media (Vector Laboratories). The number of γ-H2AX foci was visualized using a Zeiss LSM 800 laser scanning microscope (Carl Zeiss).

### Cell cycle analysis

AZD4573-treated cells were harvested, fixed in 70% ethanol, and stored at −20 ℃. After 24 h, cells were incubated with 10 μg/mL RNase A (Sigma Aldrich) at 37 ℃ for 20 min and treated with 10 μg/mL propidium iodide (Sigma Aldrich), and their DNA contents (10,000 cells) were determined using fluorescence activated cell sorting (FACS) Calibur flow cytometer (BD Biosciences).

### BrdU labeling

Before cell harvest, cells were treated with 20 mM BrdU for 30 min. Cells were then fixed in 70% ethanol and stored at – 20 ℃. DNA denaturation was performed in 4 M HCl for 20 min at room temperature, and cells were resuspended in phosphate/citric acid buffer. To investigate the level of BrdU incorporation, FITC-conjugated anti-BrdU antibodies (BD Biosciences, #347580) were used, and then cells were treated with 7-AAD (BD Biosciences). Fluorescence was measured using a FACS Calibur flow cytometer (BD Biosciences).

### Comet assay

Following AZD4573 treatment for 48 h, cells were collected and rinsed in PBS. A total of 1 × 10^6^ cells/mL were combined with 1% low melting agarose (LMAgarose) at a ratio of 1:10 and immediately pipetted onto slides. An alkaline comet assay was conducted using a Trevigen Comet Assay Kit (Trevigen, Gaithersburg, MD, USA, #4250–050-K) according to the manufacturer’s protocols. Images were taken with a Zeiss LSM 800 laser scanning microscope (Carl Zeiss), and tail intensities were measured with the Comet assay IV program (Andor Technology, Belfast, Northern Ireland, UK).

### Apoptosis assay

Quantification of apoptotic cells was determined by an Annexin V-fluorescein isothiocyanate (FITC)/propidium iodide (PI) detection kit (BD Pharmingen, #556547) according to the manufacturer’s instructions, with a minor modification on temperature. Cells were harvested after drug treatments and resuspended in a binding buffer, followed by staining with Annexin V-FITC and PI solution at 37 ℃ in the dark to improve staining efficiency. Analyses were then performed using a FACS Calibur flow cytometer (BD Biosciences).

### siRNA knock-down system

Small interfering RNAs (siRNAs) targeting CDK9, DDX25, and a non-targeting control siRNA were synthesized by Genolution (Seoul, Republic of Korea). Cells were seeded and transfected with 80 nM siRNA using Lipofectamine 3000 (Invitrogen) in Opti-MEM reduced-serum medium (Gibco), following the manufacturer’s instructions. After 8 h, the transfection medium was replaced with serum-free RPMI-1640 medium, and cells were incubated for an additional 40 h. Cells were harvested 48 h post-transfection for protein analysis, and knockdown efficiency was confirmed by Western blotting.

### Whole transcriptome sequencing (WTS)

RNA was extracted using the RNeasy Mini kit (QIAGEN, Hilden, Germany, #74140) according to the manufacturer’s instructions. For RNA library preparation, the TruSeq RNA kit (Illumina, San Diego, CA) was used for SK-BR-3, HCC70, HCC1937, and T47D cells, while the TruSeq RNA Sample Prep Kit v2 (Illumina) was employed for ZR-75–1 and HCC1428 cells. Sequencing was performed using the Illumina HiSeq 2000 platform with paired-end reads of 101 base pairs. Sequence alignment and assembly of SK-BR-3, HCC70, HCC1937, and T47D cells were conducted using TopHat version 2.0.13 and Cufflinks version 2.2.1 programs. We used HISAT2 version 2.1.0 and StringTie version 2.1.3b programs for ZR-75–1 and HCC1428 [40–45]. The human genome (GRch37/hg19) was used for reference. Raw read counts were transformed into fragments per kilobase of transcript per million (FPKM) in each dataset, and quantile normalization was performed after merging the two datasets.

### Immunoprecipitation

Cells were plated on dishes, treated with AZD4573. After 10 min, the cells were harvested, and 1 mg of total protein from the cell lysates was extracted. 1 mg of total protein was incubated with anti-S9.6 antibody and Protein A/G plus agarose (Santa Cruz biotechnology, #sc-2003) and gently shaken. The precipitates were washed with cold lysis buffer and resolved with SDS-PAGE, after which they were subjected to Western blot analysis.

### Immunohistochemistry

The 4 µm-thick tissue sections from individual paraffin-embedded formalin-fixed xenograft tumor samples were deparaffinized and dehydrated. Immunohistochemistry (IHC) was performed using the following antibodies: Ki-67 (Thermo Fisher Inc., #MA5-14520) at a dilution of 1:300, S9.6 (Kerafast, Boston, MA, USA, #ENH001) at a dilution of 1:500 and the anti-rabbit polyclonal antibody against DDX25 (Atlas antibodies, Bromma, Stockholm, Sweden, #HPA020137) at a dilution of 1:200.The immunostained slides were scanned using Aperio AT2 (Leica, Buffalo Grove, IL, USA). Scanned IHC images acquired at 40 × magnification were used for the analysis. The analysis utilized QuPath version 0.3.2 [46, 47]. Two researchers (M.L. and J.K.) independently designated representative regions for image analysis. The positive cell percentages were assessed using a single threshold option with a cutoff of 0.4.

### In vivo study

In vivo xenograft experiments were conducted using six-week-old female BALB/c nude mice purchased from Orient Bio (Seongnam, Republic of Korea). All animals were maintained under specific pathogen-free (SPF) conditions with controlled temperature and humidity and a 12-h light/dark cycle. Mice were housed in groups of five per cage and provided with food and water ad libitum. All animal procedures were approved by the Institutional Animal Care and Use Committee (IACUC) of the Institute of Biomedical Science, Seoul National University, under the approval number 23–0379-S1A0, and were performed according to relevant guidelines and regulations.

### Statistical analysis

Statistical analyses were performed using Sigma Plot (version 10.0) and R (version 4.4.0). A two-sided student’s *t-test* was used when appropriate. The results are expressed as the mean standard deviation, or standard error. A *p*-value less than 0.05 was considered statistically significant.

## Results

### AZD4573 has a cytotoxic effect on breast cancer cells regardless of CDK9 or MCL1 expression level

**Fig. 1:**
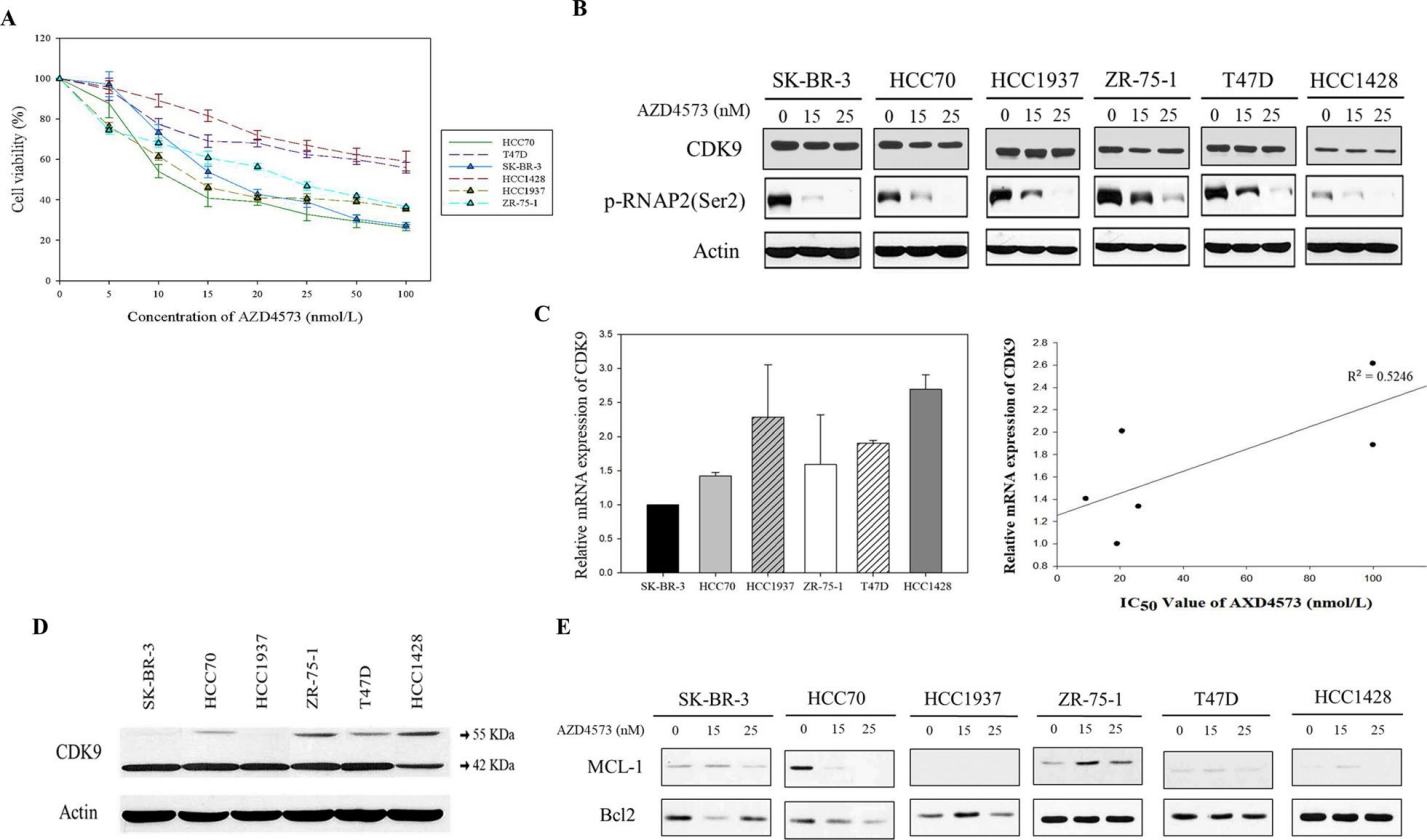
AZD4573 has a cytotoxic effect on breast cancer cells regardless of CDK9 or MCL-1 expression. **A** The cytotoxic effect of AZD4573 in breast cancer cells was measured using the MTT assay. Results were presented as a graph with standard error bars. **B** AZD4573 effectively inhibited CDK9 in breast cancer cell lines. Actin was used as a lading control. **C** Correlation between the IC50 value of AZD4573 and the mRNA expression of CDK9 is not statistically significant. The correlation was measured by the R value in a scatter plot of a linear regression line graph based on points. **D** CDK9 protein expressions were shown by Western blot. **E** Significant association between AZD4573 sensitivity and expression levels of MCL1 was not observed

The effect of AZD4573 on breast cancer cells was evaluated by MTT assay (Fig. 1A and S1A). AZD4573 showed diverse efficacy, with IC_50_ values ranging from 9.16 to > 100 nmol/L in breast cancer cell lines (Table 1). Based on these values, we divided the cell lines into two groups: AZD4573-sensitive and less-sensitive cells. SK-BR-3, HCC70, HCC1937, and ZR-75–1 cells had IC_50_ values of less than 25 nmol/L, thus selected as sensitive cells. T47D and HCC1428 cells were characterized as less-sensitive cells with IC_50_ values higher than 100 nmol/L. To confirm the CDK9 inhibition by AZD4573, phosphorylation of RNAP2, a direct target of CDK9, was evaluated by Western blot. AZD4573 treatment resulted in decreased phosphorylation of RNAP2 in both sensitive and less-sensitive cell lines, suggesting that AZD4573 successfully inhibits CDK9 activity in breast cancer cell lines (Fig. 1B and S1B). Then, mRNA and protein levels of *CDK9* in each cell were analyzed (Fig. 1B–D), where no correlation was observed when compared to the cytotoxicity induced by AZD4573. Furthermore, we evaluated the mRNA expression levels of *CDK9* and *MYC*, considering that *MYC* is known to be transcriptionally regulated by *CDK9* [48]*.* There was no significant difference in *CDK9* and *MYC* expression levels among the investigated breast cancer cells at the mRNA level (Fig. S1C and S1D). These data indicate that the cytotoxic effect of AZD4573 in breast cancer cell lines is less likely dependent on the level of *CDK9* expression. Previous studies revealed that CDK9 inhibitors induce apoptotic cell death, reducing the expression level of MCL1 in hematological cancers. To identify whether AZD4573 leads to apoptosis by down-regulation of *MCL1* in breast cancer cells, the expression level of *MCL1* was evaluated. After the treatment with AZD4573, reduced expression of *MCL1* was observed only in HCC70 cells, however, notable differences in *MCL1* expression were not seen in other sensitive cells (Fig. 1E). In addition, protein expression levels of apoptotic molecules were analyzed in breast cancer cells, but there were no differences according to the sensitivity of AZD4573 in breast cancer cells (Fig. 1A and S1D). Collectively, these results suggest that the anti-tumor effects of AZD4573 in breast cancer cells are regulated by mechanisms other than mere down-regulation of *CDK9* or *MCL1*.

**Table 1:**
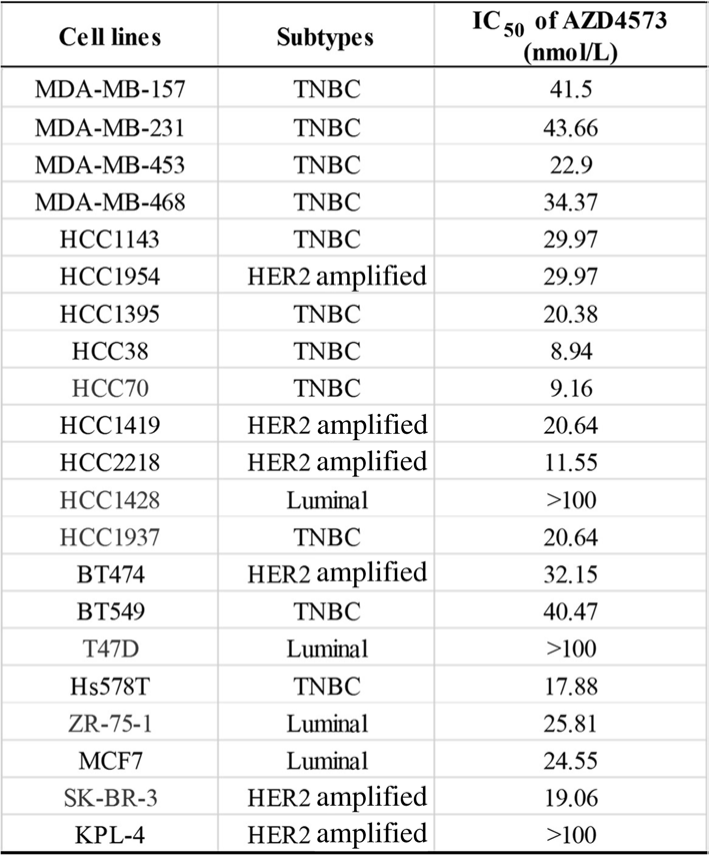
IC_50_ values of AZD4573 in breast cancer cells

### AZD4573 induces T-R conflicts during S-phase

**Fig. 2:**
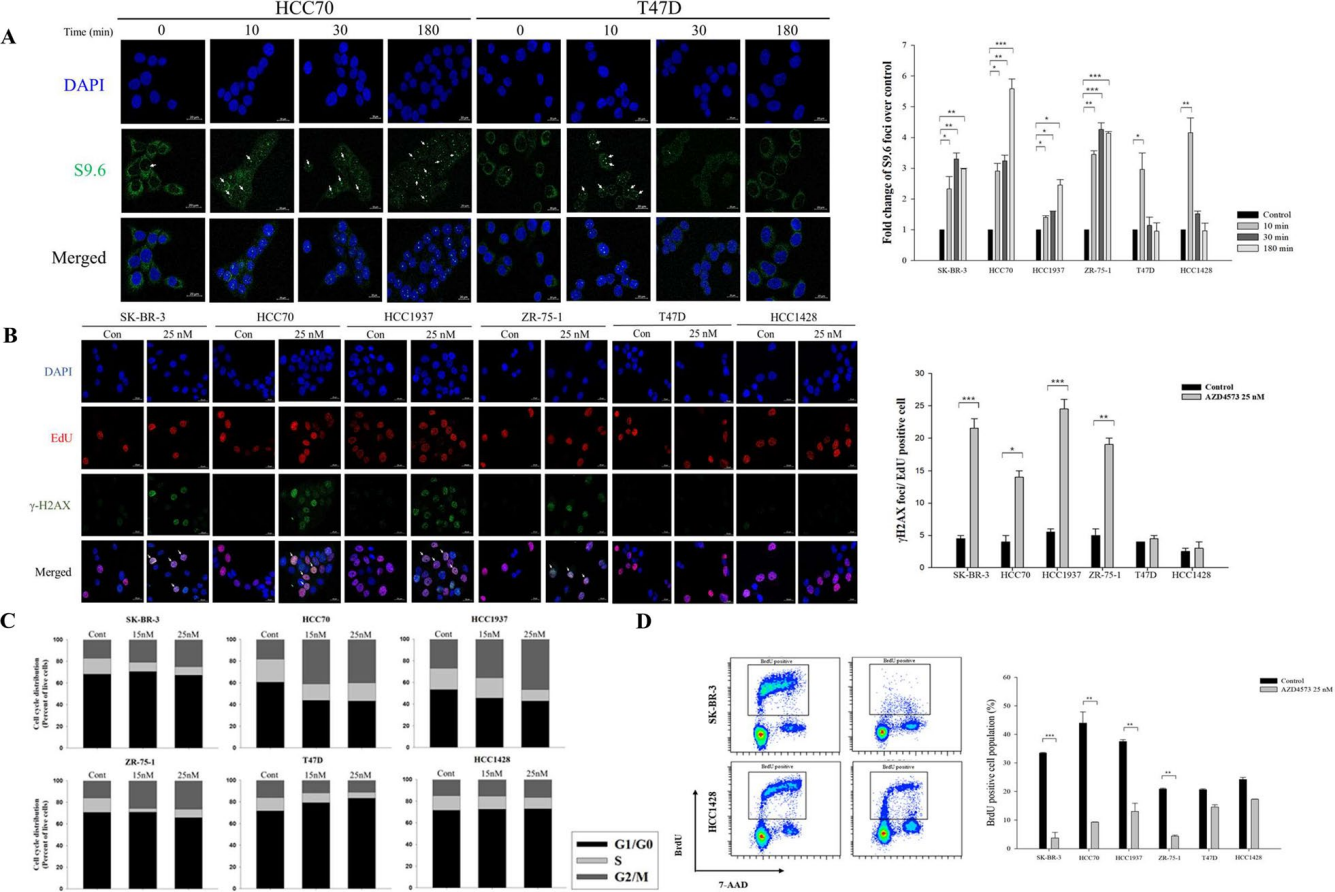
AZD4573 induces T-R conflicts during replication. **A** T-R conflicts persisted in sensitive cells, whereas they were rapidly resolved in less-sensitive cells. Representative images from immunofluorescence staining of T-R conflicts in HCC70 cells, sensitive cells to AZD4573, and in T47D cells, less-sensitive cells to AZD4573. Microscopic images of stained cells were acquired at × 40 magnification. The number of S9.6 foci/cells is quantified using Sigmaplot and presented in a bar graph (means ± SE). Experiments were conducted in duplicate, and the results were repeated. At least 100 nuclei were analyzed for each experiment. **B** AZD4573 leads to DNA damage in replicating cells. γ-H2AX foci formation in S-phase was conducted via EdU-incorporation assay. Microscopic images of stained cells were acquired at × 40 magnification. The number of γ-H2AX foci was quantified by Sigmaplot and presented as a bar graph (means ± SE). Experiments were conducted in duplicate, and the results were repeated. At least 80 EdU-positive cells were analyzed for each experiment. **C** Decreased S-phase proportions were observed in sensitive cells. Cells were treated with indicated doses of AZD4573 for 24 h. Stacked bar graph represents the distribution of cells in G1, S, and G2/M phases, analyzed exclusively from the live cell population. Columns represent the average of three independent experiments. **D** Sensitive cells exhibited a reduced S-phase proportion following treatment with 25 nmol/L AZD4573 for 48 h, S-phase progression was examined by BrdU-labeling. BrdU positive cell population is quantified by Sigmaplot and presented in a bar graph (means ± SE). *p-value < 0.05, **p-value < 0.01, ***p-value < 0.001. The scale bars represent 20 μm

Recent studies have shown that the inhibition of CDK9 is critical for the paused state of RNAP2 near the promoter region, which induces T-R conflicts. Mechanistically, T-R conflicts aberrantly form DNA-RNA hybrid structures that serve as barriers for DNA polymerase progression[9, 21, 49]. Therefore, we hypothesized that AZD4573 leads to a collision between the transcription and replication machinery and promotes replication stress. To examine whether AZD4573 triggers T-R conflicts, an immunofluorescence assay was conducted using anti-S9.6 antibodies that recognize RNA–DNA hybrids. The number of S9.6 foci at 10 min in SK-BR-3, HCC70, HCC1937, and ZR-75–1 breast cancer cells with AZD4573 increased by 1.4–3.5 fold compared to cells treated with DMSO. Furthermore, T-R conflicts remained for 3 h in sensitive breast cancer cells, while they disappeared immediately within 30 min in less-sensitive cells (Fig. 2A and S2A). To examine the effect of T-R conflicts on DNA damage of replicating cells, the number of γ-H2AX foci co-localized with EdU was analyzed. In AZD4573-sensitive breast cancer cells, the numbers of γ-H2AX foci in EdU-positive cells were increased by 3.5–4.8 fold within 3 h following AZD4573 treatment compared to controls (Fig. 2B). We also conducted cell cycle analysis to investigate the influence of T-R conflict on the overall cell cycle. A decreased proportion of cells in the S-phase in sensitive cells was confirmed. On the contrary, a dose-dependent increase in the cells in the G1 phase was observed in less-sensitive cells. (Fig. 2C and S2B). To further verify the reduced proportion of the S-phase cells, a BrdU assay was performed, and the results validated that the S-phase cell proportions indeed decreased in sensitive cells (Fig. 2D and S2B). Our findings demonstrate that AZD4573 induces T-R conflicts, which serve as a source of replication stress and may potentially lead to DNA damage.

### T-R conflicts lead to the accumulation of DNA damage and caspase-3-dependent apoptosis

**Fig. 3:**
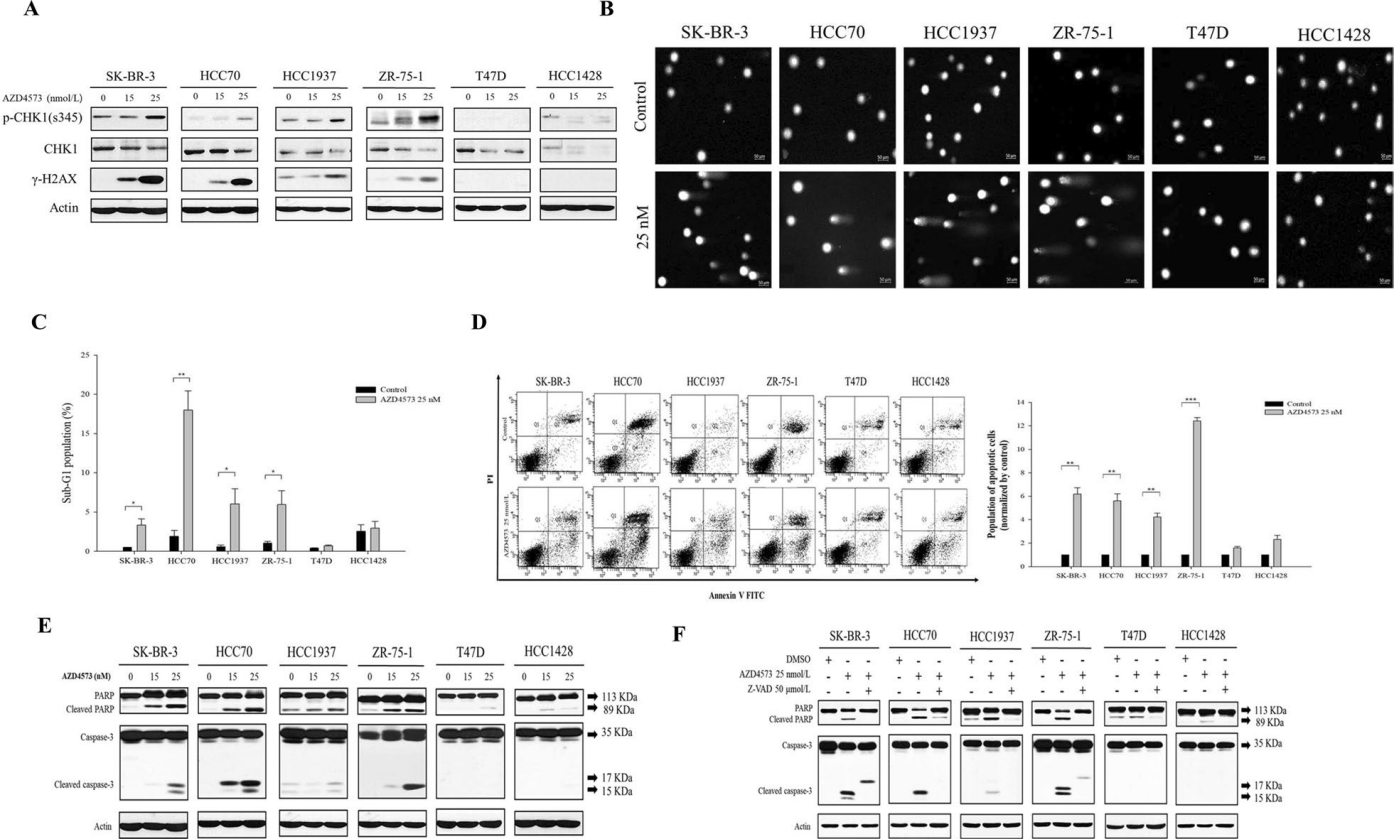
T-R conflicts result in the accumulation of DNA damage leading to caspase-3-dependent apoptosis. **A** Expression levels of DNA damage response molecules in breast cancer cells were increased in sensitive cells following the increasing doses (0, 15, 25 nmol/L) of AZD4573 treatment for 48 h. Actin was used as a loading control. **B** Accumulation of DNA damage was observed in breast cancer cells via the comet assay. Cells were incubated with 25 nmol/L of AZD4573 for 48 h. **C** sub-G1 population was increased in sensitive cells. Population of sub-G1 is quantified by Sigmaplot and presented in a bar graph (means ± SE). **D** Cells that are undergoing apoptosis were increased in sensitive cells to AZD4573. Representative scatter plots of PI (y-axis) vs. Annexin-V (x-axis). The population of apoptotic cells was presented as the mean ± SD of triplicate experiments. **E** Increased expression levels of cleaved PARP and cleaved caspase-3 were observed in sensitive cells by Western blot. Cells were treated with increasing doses (0, 15, 25 nmol/L) of AZD4573 for 48 h. **F** Complete activation of caspase-3 and the increased expression of cleaved PARP were not observed in sensitive cells treated with AZD4573 and Z -VAD FMK Cells were treated 25 nmol/L AZD4573 alone or with 50 μmol/L of Z-VAD, a pan-caspase inhibitor, for 48 h

To verify that increased T-R conflicts by AZD4573 induce DNA damage accumulation, the expression level of γ-H2AX and checkpoint kinase 1 (CHK1) was assessed. After AZD4573 treatment for 48 h, the levels of γ-H2AX expression and chk1 phosphorylation were increased in sensitive cells in a dose-dependent manner (Fig. 3A). Concordant with this data, comet tails, indicating DNA damage, were significantly increased in sensitive cells (Fig. 3B). Cells respond to endogenous DNA damage by activating DNA damage response (DDR) to maintain genomic stability. However, if cells are exposed to severe DNA damage, DDR triggers apoptosis [50, 51]. To assess whether AZD4573 induces apoptosis, an analysis of the sub-G1 population was conducted. After treatment with AZD4573, the sub-G1 population increased by 3.3—9.5 folds compared to control in sensitive cells (Fig. 3C). Additionally, we carried out an Annexin V assay. The population of SK-BR-3, HCC70, HCC1937, and ZR-75–1 cells that are undergoing apoptosis was increased by 4.2–12.4 folds following AZD4573 treatment in sensitive cells, but there were no significant changes in less-sensitive cells (Fig. 3D). Increased protein levels of cleaved PARP also demonstrated this induction of apoptosis and cleaved caspase-3 in sensitive cells (Fig. 3E). Moreover, to identify whether apoptosis was specifically regulated by caspase-3 activation, we evaluated the level of cleaved PARP and caspase-3 in AZD4573-treated breast cancer cells and cells treated with 50 µM of a pan-caspase inhibitor, Z-VAD-FMK [52]. The expression level of cleaved PARP and cleaved caspase-3 was increased in sensitive cells following treatment with AZD4573. However, complete activation of caspase-3 and the increased expression of cleaved PARP were not observed in sensitive cells treated with AZD4573 and Z-VAD-FMK (Fig. 3F). These findings indicate that AZD4573 induces apoptosis following the accumulation of DNA damage in sensitive cells.

### Knockdown of *CDK9* results in cell growth inhibition, the accumulation of DNA damage, and increased T-R conflicts

**Fig. 4:**
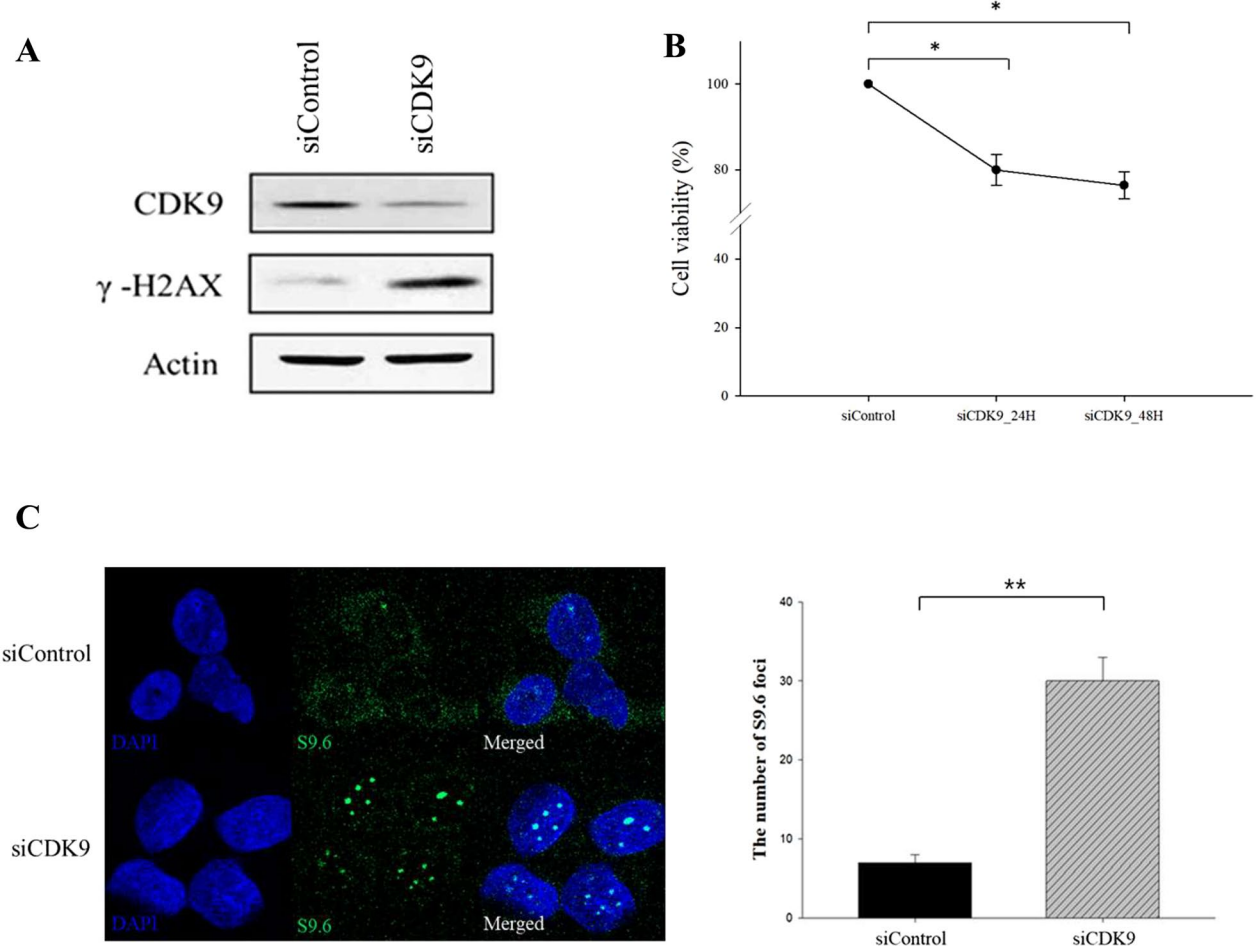
Knock-down of CDK9 results in cell growth inhibition, the accumulation of DNA damage, and increased T-R conflicts. **A** siRNA-mediated reduction of CDK9 expression and increased DNA damage was confirmed in HCC1937 cells. Actin was used as a loading control. **B** Viability of CDK9 down-regulated HCC1937 cells was measured by MTT assay. Results were presented as means with standard error. **C** T-R conflicts were induced in the si*CDK9* transfected HCC1937 cells. Representative images from immunofluorescence staining of T-R conflicts in si*CDK9* transfected HCC1937 cells. The number of S9.6 foci obtained in triplicate is quantified using Sigmaplot and presented in a bar graph (means ± SE). At least 100 nuclei were analyzed for each experiment. *p-value < 0.05

To validate whether the antitumor effect of AZD4573 is attributed specifically to the target itself, we knocked down *CDK9* using siRNA oligonucleotides in HCC1937 cells. The expression level of *CDK9* was decreased, and γ-H2AX level was increased after si*CDK9* transfection (Fig. 4A). Growth inhibition was also observed in CDK9-knockdown HCC1937 cells (Fig. 4B). In addition, consistent with prior observation, T-R conflicts were increased by 4.3 fold in the si*CDK9*-transfected HCC1937 cells compared to the control (Fig. 4C). These results suggest that AZD4573 exerts anti-tumor effects on breast cancer cells by specifically targeting CDK9.

### Nuclear translocation of DDX25 helicase promotes the resolution of T-R conflicts

**Fig. 5:**
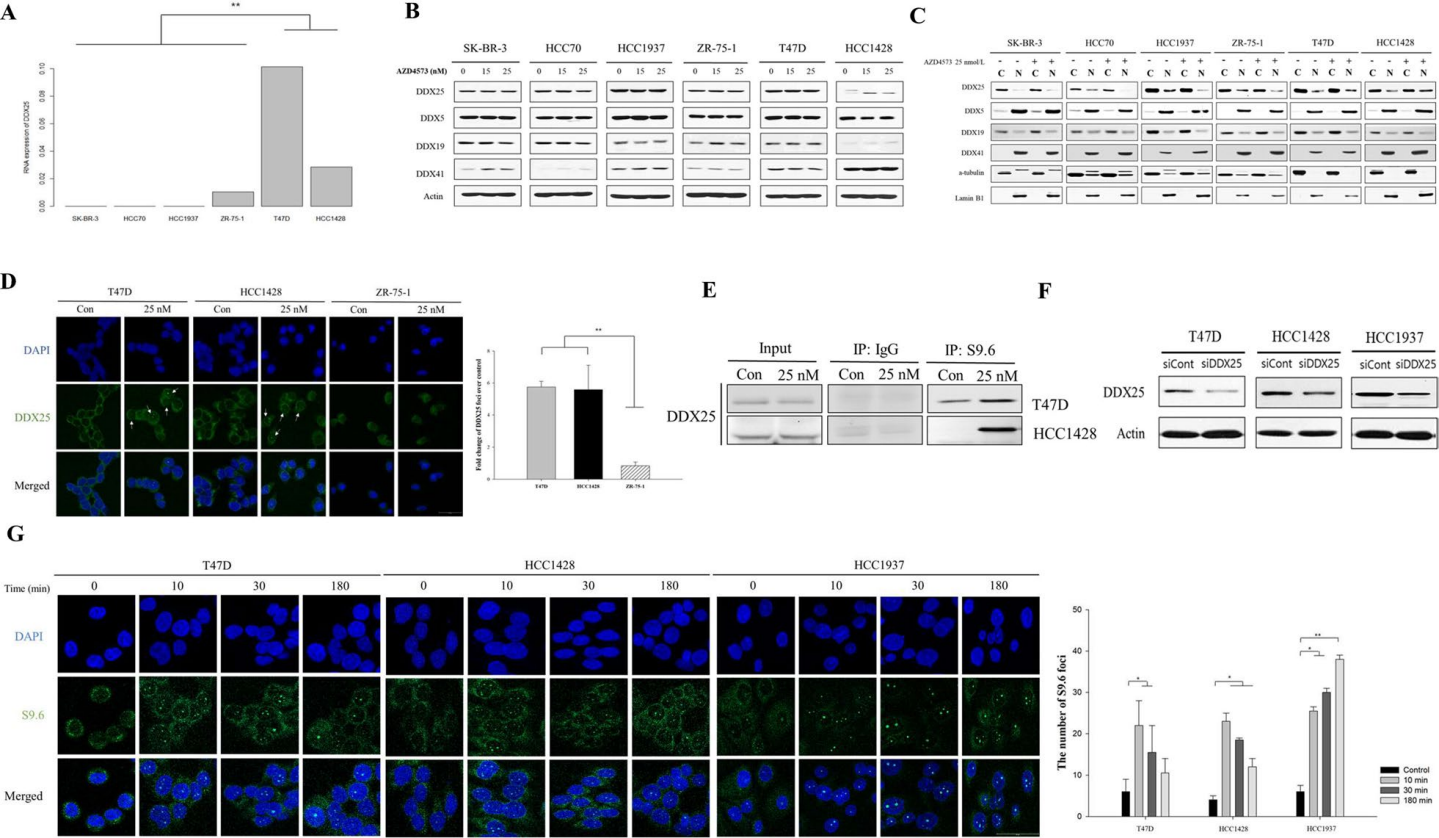
DDX25 helicase is involved in T-R conflicts resolution through nuclear translocation. **A** Significant differences in the expression levels of DDX25 was observed according to the drug sensitivity. **B** Expression levels of DDX helicases remained unchanged in breast cancer cells. Actin was used as a loading control. **C** The amount of DDX25 helicase within the nuclear subfraction was elevated in less-sensitive cells to AZD4573. Cells were treated 25 nmol/L of AZD4573 for 3 h. A-tubulin and lamin B1 were used as cytoplasmic and nuclear markers. **D** DDX25 nuclear translocation was significantly increased in less-sensitive cells to AZD4573. DDX25 helicase foci formation in the nucleus was conducted via immunofluorescence assay. The number of DDX25 helicase foci was quantified by Sigmaplot and presented as a bar graph (means ± SE). Experiments were conducted in duplicate, and results were repeated. At least 30 nuclei were analyzed for each experiment. **E** DDX25 directly interacts with T-R conflicts. Breast cancer whole extracts were immunoprecipitated with DNA-RNA hybrids (S9.6) antibody or IgG antibody. **F** siRNA-mediated reduction of *DDX25* expression was confirmed by Western blot. **G** Persistent T-R conflicts were observed in si*DDX25*-transfected T47D and HCC1428 cells. S9.6 foci formation in nucleus was conducted via immunofluorescence assay. The number of S9.6 foci was quantified by Sigmaplot and presented as a bar graph (means ± SE). *p-value < 0.05, **p-value < 0.05

We established that the persistence of T-R conflicts determines sensitivity to AZD4573 (Fig. 2A). In this regard, we postulated that helicases may be involved in the resolution of R-loop and T-R conflicts in AZD4573 less-sensitive cell lines. We analyzed WTS data to compare gene expression levels of diverse helicases across breast cancer cells. Interestingly, higher expression of *DDX25* was observed in less-sensitive cells compared to sensitive cells to AZD4573, while sensitive cell lines were nearly devoid of DDX25 expression (*p* < 0.05) (Fig. 5A). We also examined the protein expression level of DDX25 helicases and several DDX helicases involved in R-loop resolution, such as DDX5*,* DDX19, and DDX41. Unexpectedly, the expression levels of DDX helicases remained unchanged after AZD4573 treatment (Fig. 5B). We investigated whether the nuclear and cytosolic subfractions of DDX25 were altered after AZD4573 treatment. The amount of DDX25 helicase in the nuclear subfraction was increased in less-sensitive cells to AZD4573 (Fig. 5C). To validate this further, nuclear translocation of DDX25 was assessed using immunofluorescence, and a significant increase of DDX25 nuclear translocation was confirmed in less-sensitive cells, consistent with prior observations (*p* < 0.05) (Fig. 5D). Moreover, we conducted immunoprecipitation (IP) to explore whether DDX25 directly engages with T-R conflicts and contributes to their resolution. Indeed, the interaction between DDX25 and T-R conflicts was identified in T47D and HCC1428 cells (Fig. 5E). Furthermore, to assess the involvement of DDX25 in R-loop resolution in the nucleus, we employed siRNA-mediated knockdown of *DDX25* and evaluated the formation of R-loops. The T-R conflicts persisted in si*DDX25*-transfected T47D and HCC1428 cells compared to the control (Fig. 5F, G). Taken together, the nuclear translocation of DDX25 is crucial for the resolution of T-R conflicts, which influences the sensitivity to AZD4573 in breast cancer cells.

### AZD4573 induces T-R conflicts and inhibits tumor growth in the xenograft mouse model

**Fig. 6:**
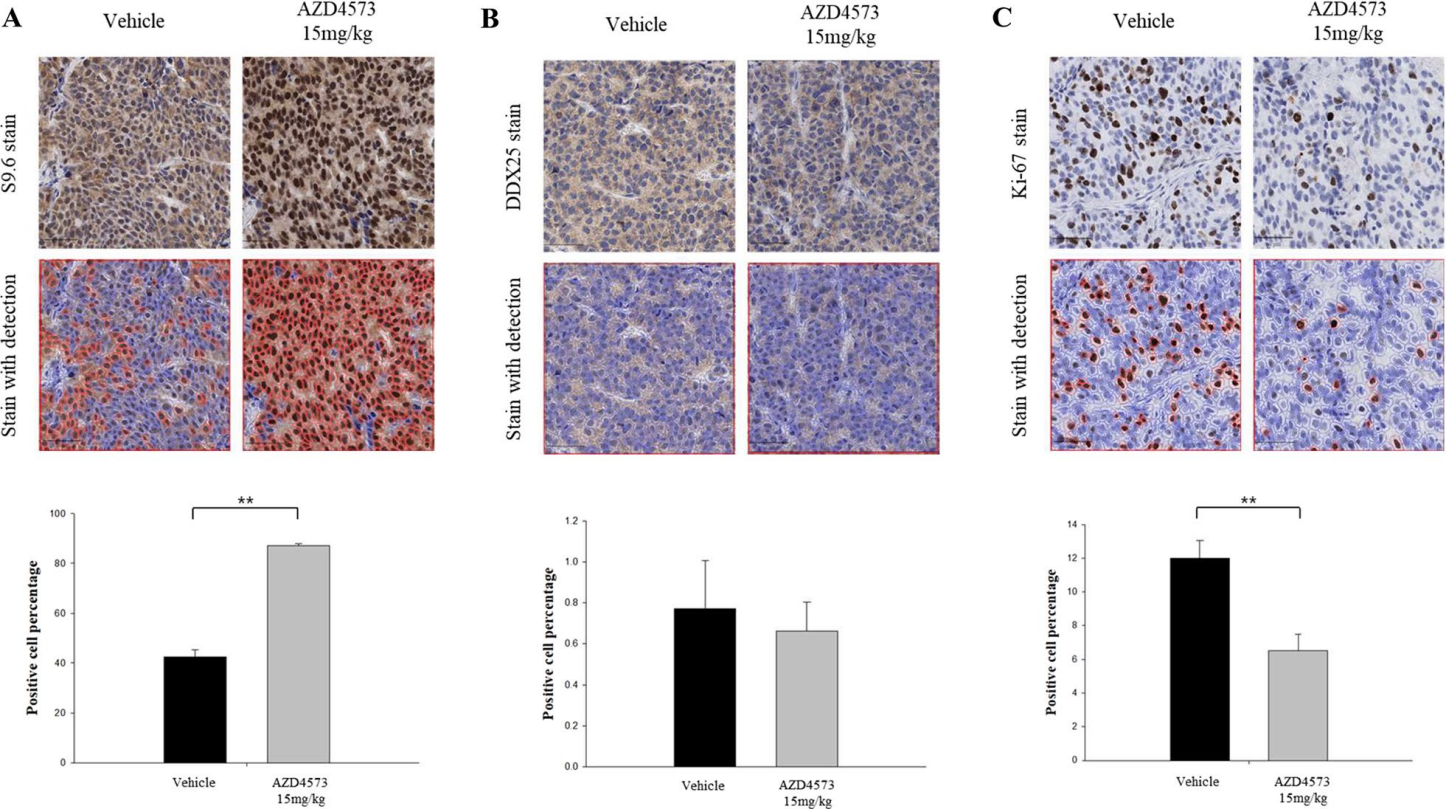
AZD4573 induces T-R conflicts and inhibits tumor growth in xenograft mouse model. Tumors were removed from the mice after the vehicle control or drug treatment ended. **A** IHC stain for T-R conflicts using S9.6 antibody showed an increase in the AZD4573-treated tumor. Microscopic images of stained cells were acquired at × 40 magnification. **B** Significant expression of DDX25 was not observed in the AZD4573-treated and control tumors. **C** Lower proliferation index measured by Ki-67 immunostaining was noted in AZD4573-treated tumors. The scale bars represent 50 μm. Analysis were conducted in duplicate and results were repeated. **p-value < 0.05

To confirm the in vitro findings in an in vivo setting, we used a mouse xenograft model of ZR-75–1 human breast cancer cells implanted in the mammary fat pad. Mice were randomly treated with vehicle alone or 15 mg/kg of AZD4573 (twice daily with a 2-hour split on 2-day on, 5-day off) for 3 weeks using intraperitoneal (IP) injection. After completion of the 3-week treatment, tumors were excised and subjected to IHC (Fig. S3A). Tumor tissues from the AZD4573-treated mouse xenograft model showed induction of T-R conflicts in the nucleus, represented by higher percentages of S9.6-positive cells compared to the control, with 87% in the AZD4573-treated mouse and 43% in the control mouse (*p* < 0.05) (Fig. 6A). On the other hand, no significant differences in nuclear expression of DDX25 helicase between tumor tissues from mouse treated with AZD4573 and control (Fig. 6B). Consistent with our in vitro findings, treatment with AZD4573 was found to increase T-R conflicts, while DDX25 failed to translocate into the nucleus in a xenograft mouse model. AZD4573 treatment resulted in a substantial reduction in tumor growth compared to vehicle treatment (Fig. S3B). Ki-67 IHC was evaluated to assess the extent of anti-proliferative activity of AZD4573. Tumor tissues from mice treated with AZD4573 showed lower Ki-67 expression, with 6.5% in the AZD4573-treated mouse and 12% in the control mouse (*p* < 0.05) (Fig. 6C). Collectively, we could verify not only the interaction between T-R conflicts and DDX25 helicases, but also the impact they have on cell proliferation in the xenograft model.

## Discussion

CDK9 is a crucial regulator of transcriptional progression from initiation to elongation [3, 10, 14]. The effects of CDK9 have been studied in hematologic malignancies, and many of them have suggested the downregulation of master transcription factor MYC or upregulation of anti-apoptotic molecules such as MCL1 and BCL2. However, few studies have investigated the anti-tumor effect of AZD4573 on solid tumors. Moreover, previous studies have suggested a potential prognostic role of CDK9 expression in breast cancer. In particular, high CDK9 expression has been associated with worse overall survival (OS) in basal-like and luminal B subtypes based on transcriptomic analyses using publicly available datasets. Higher CDK9 expression was found to correlate with shorter OS, suggesting its significant role in breast cancer progression and poorer prognosis [53]. Based on these findings, we hypothesized that CDK9 could be a potential therapeutic target. This led us to investigate the role of CDK9 in breast cancer, focusing on its potential for therapeutic intervention.

Consistent with the previous studies, we observed that AZD4573 induced apoptotic cell death. However, the apoptotic cell death did not correlate with the levels of MCL1 expression in breast cancer cells (Fig. 1E), suggesting that AZD4573 induces tumor cell death through another mechanism in breast cancer cells.

CDK9 permits the release of paused RNAP2 from the proximal promoter region and prevents collision between transcription and replication. Previous studies have shown that CDK9 inhibition is a primary source of T-R conflicts and genome instability. Collisions between transcription and replication result in the R-loop structures, DNA-RNA hybrids formed by RNA transcripts and template DNA strands, leading to topological stress. T-R conflicts can slow down replication forks, causing replication stress. Furthermore, the other DNA strand is exposed as single-strand DNA, resulting in double-strand breaks following transcription-coupled nucleotide excision repair. Thus, T-R conflicts are considered a critical mechanism of inducing replication stress and DNA damage [9, 13, 18, 54]. Therefore, we hypothesized that apoptotic cell death following AZD4573 treatment occurred because of the DNA damage accumulation caused by increased T-R conflicts. We confirmed that AZD4573-induced T-R conflicts lead to the accumulation of DNA damage in breast cancer cells. Interestingly, we found that T-R conflicts started to appear both in sensitive and less-sensitive cells to AZD4573 within 10 min. In contrast, prolonged T-R conflicts after 3 h were observed only in sensitive cells. T-R conflicts disappeared immediately within 30 min in less-sensitive cells. Indeed, the results showed that DNA damage caused by T-R conflicts was accumulated in sensitive cells, but not in less-sensitive cells (Fig. 2A¸B). These findings suggest that proper resolution of T-R conflicts is a crucial mechanism to avoid cell death after AZD4573 treatment.

Subsequently, we focused on identifying specific molecular regulators responsible for T-R conflict resolution. By analyzing WTS data, we identified a significant difference in the expression level of DDX25 helicase between sensitive and less-sensitive cells (Fig. 5A). We validated that DDX25 helicase is involved in regulating T-R conflicts upon AZD4573 treatment. According to previous studies, it has been shown that nuclear translocation of DDX helicases is crucial for R-loop resolution [29]. Notably, the nuclear expression level of DDX25 correlated with AZD4573-induced cell death (Fig. 5D). Considering the essential role of resolving T-R conflicts in maintaining cell viability, we propose that DDX25, the RNA helicase may serve both as a functional modulator of T-R conflicts resolution and a predictive biomarker for AZD4573 response in breast cancer. The precise mechanism of DDX25 translocation and its regulation under replicative stress conditions remains to be elucidated.

In addition to its impact on transcriptional regulation and replication stress, CDK9 inhibition may also affect DNA repair pathways. Recent studies have shown that CDK9 could downregulate the expression of BRCA1, a key player in homologous recombination (HR) repair, and prevent the recruitment of BRCA1 to DNA damage sites [55, 56]. This finding provides a mechanistic rationale for combining CDK9 inhibitors with other DNA-damaging agents or DDR inhibitors.

Among these, poly (ADP-ribose) polymerase (PARP) inhibitors have emerged as promising therapeutic agents. PARP is a key enzyme that senses DNA damage and initiates the repair of DNA single strand breaks (SSBs), which increases DNA double strand breaks (DSBs) through collision of unrepaired SSB with DNA replication fork, inducing replication fork collapse [57]. Thus, inhibition of PARP induces overwhelming DSBs in cells with mutations in *BRCA1/2* based on synthetic lethality [58–60]. In previous studies, the synergetic effect of olaparib, a PARP inhibitor, combined with CDKI-73, a CDK9 inhibitor, was reported in BRCA1-proficient ovarian cancer cells; the combination resulted in a reduction of BRCA1 expression and recruitment to DNA damage sites [61]. These studies indicate that CDK9 inhibition could have a substantial impact on the HR pathway through its effects on BRCA1 expression. In addition, down-regulated BRCA1 expression in breast cancer cells was observed following AZD4573 treatment in this study (Fig. S4). Taken together, we propose that CDK9 not only regulates transcriptional elongation but also plays a broader role in maintaining genomic integrity by preventing T-R conflicts. Moreover, CDK9 inhibitors can be ideal candidates for combination with other DDR inhibitors and DNA-damaging agents.

## Conclusion

We have demonstrated that CDK9 inhibitor AZD4573 exhibits the anti-tumor effect on breast cancer cells, inducing cell death through the accumulation of DNA damage caused by persistent T-R conflicts. Furthermore, the nuclear expression of DDX25 may serve as a predictive biomarker for the response to AZD4573 treatment in breast cancer. These findings offer promising prospects for developing novel therapeutic strategies and provide a biomarker for predicting drug response in breast cancer.

## Supplementary Information

Additional file 1

## Data Availability

The datasets generated or analysed during the current study are available from the corresponding author upon reasonable request.

## Abbreviations

BLM: Bloom syndrome
CDK: Cyclin-dependent Kinase
CHK1: Checkpoint kinase 1
CTD: C-terminal domain
DDR: DNA damage response
DDX helicase: Dead-box helicase
DSB: Double strand break
FACS: Fluorescence activated cell sorting
FANC: Fanconi anemia complementation group
FPKM: Fragments per kilobase of transcript per million
IC50: Half maximal inhibitory concentration
IFA: Immunofluorescence
IHC: Immunohistochemistry
IP: Immunoprecipitation
NELF: Negative- elongation factor
PARP: Poly (ADP-ribose) polymerase
RNAP2: RNA polymerase-II
SETX: Senataxin
SRSF1: Serine/arginine-rich splicing factor1
SSB: Single strand break
TOP1: Topoisomerase 1
T-R conflict: Transcription-replication conflict
TREX: Transcription-export
WRN: Werner syndrome
WTS: Whole transcriptome sequencing
HR: Homologous recombination
