# Supplementary material for: Cyclin dependent kinase 9 inhibitor induces transcription-replication conflicts and DNA damage accumulation in breast cancer
Source: Cancer Cell Int. 2025 Jul 25;25:282. doi: 10.1186/s12935-025-03897-6 (PMC12297744; doi:10.1186/s12935-025-03897-6)

Supplementary figure 1

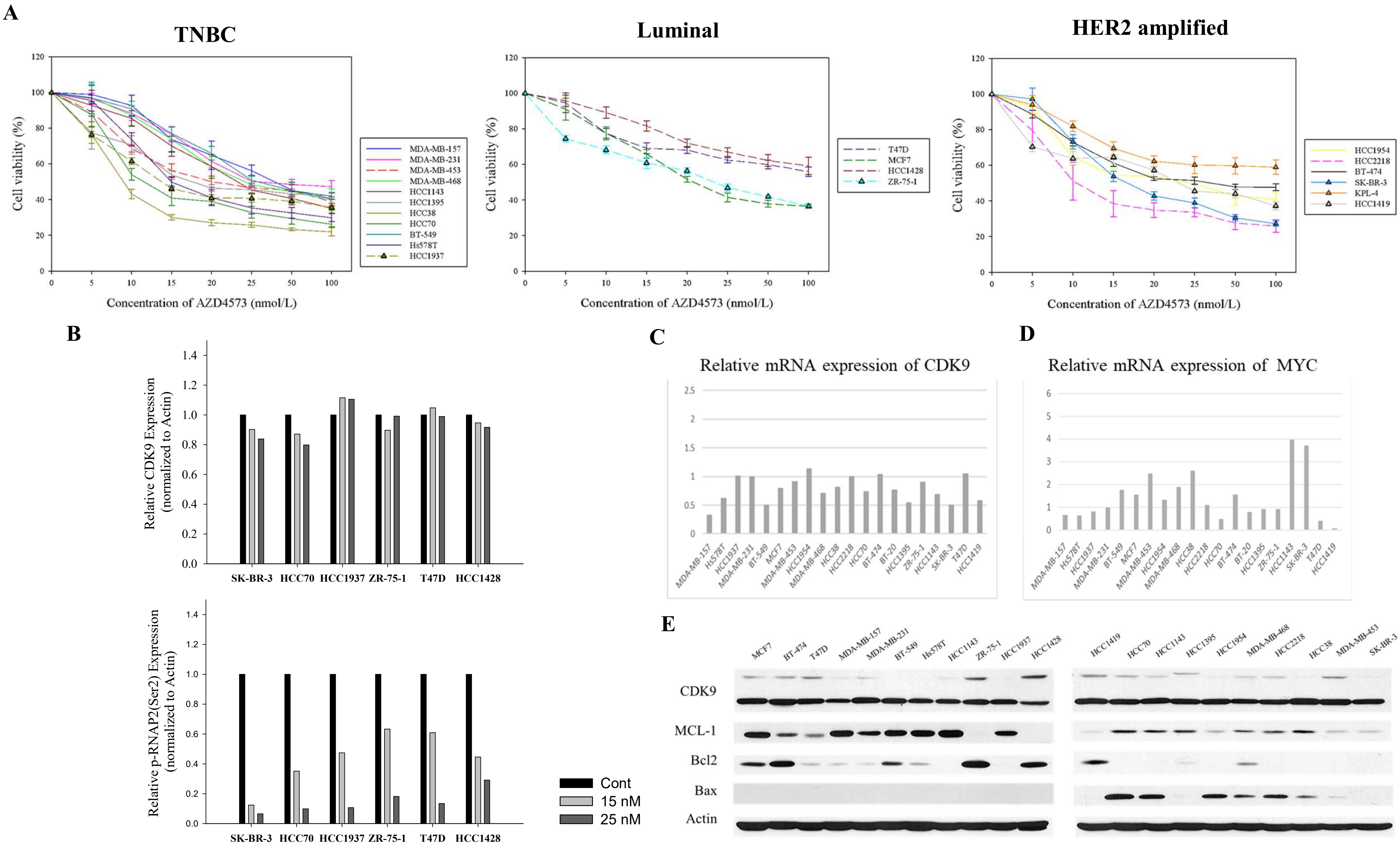

Supplementary figure 2

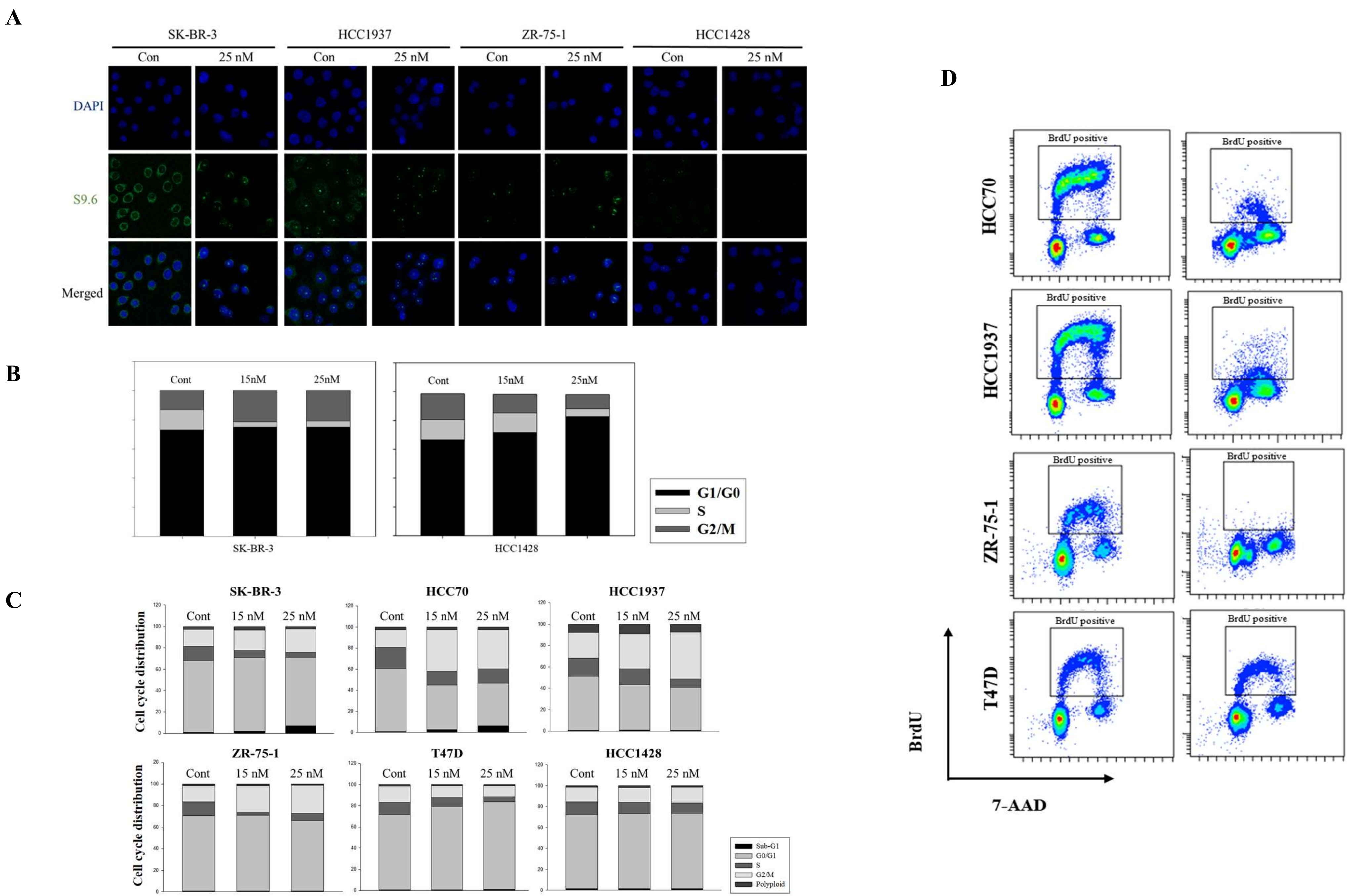

Supplementary figure 3

A

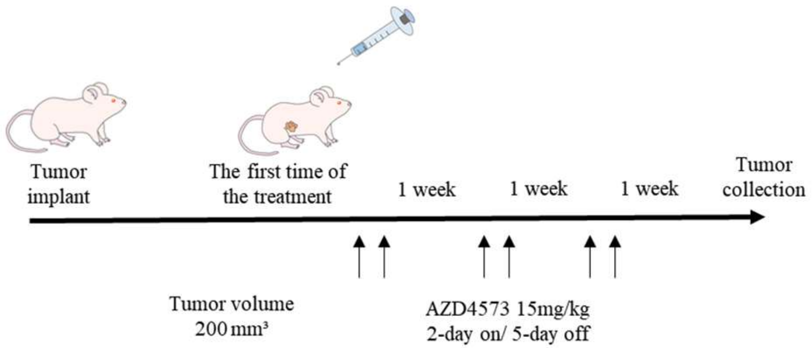

B

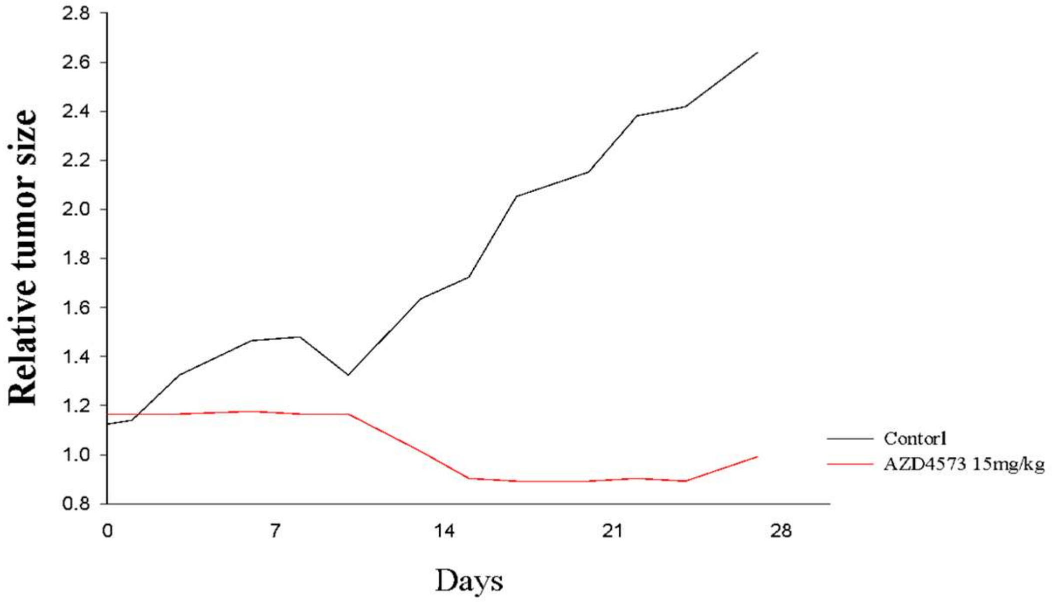

Supplementary figure 4

A

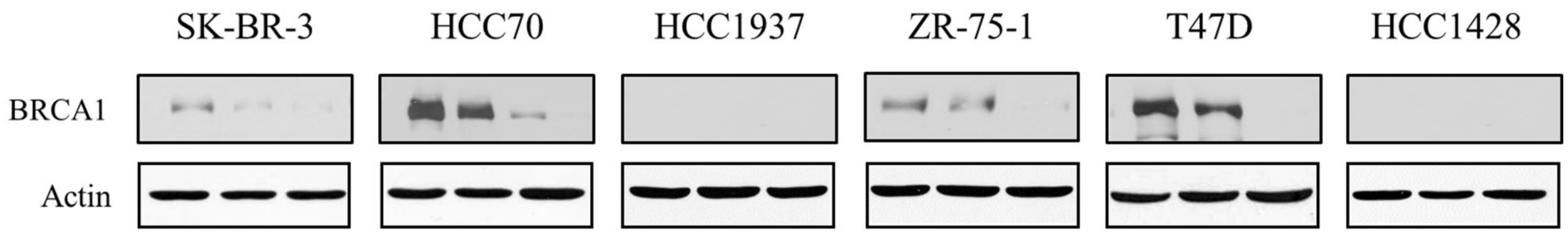

Supplement: Supplementary file 1 — Additional file 1 [file 12935_2025_3897_MOESM1_ESM.pdf]
